# PCBP2 Enhances the Antiviral Activity of IFN-α against HCV by Stabilizing the mRNA of STAT1 and STAT2

**DOI:** 10.1371/journal.pone.0025419

**Published:** 2011-10-11

**Authors:** Zhongshuai Xin, Wei Han, Zhiqiang Zhao, Qing Xia, Bin Yin, Jiangang Yuan, Xiaozhong Peng

**Affiliations:** 1 National Laboratory of Medical Molecular Biology, Institute of Basic Medical Sciences, Chinese Academy of Medical Sciences and Peking Union Medical College, Beijing, China; 2 Division of Biochemical and Gene Engineering Medicines, National Institute for Food and Drug Control, Beijing, China; 3 National Center of Biomedical Analysis, Beijing, China; University of Kansas Medical Center, United States of America

## Abstract

Interferon-α (IFN-α) is a natural choice for the treatment of hepatitis C, but half of the chronically infected individuals do not achieve sustained clearance of hepatitis C virus (HCV) during treatment with IFN-α alone. The virus can impair IFN-α signaling and cellular factors that have an effect on the viral life cycles. We found that the protein PCBP2 is down-regulated in HCV-replicon containing cells (R1b). However, the effects and mechanisms of PCBP2 on HCV are unclear. To determine the effect of PCBP2 on HCV, overexpression and knockdown of PCBP2 were performed in R1b cells. Interestingly, we found that PCBP2 can facilitate the antiviral activity of IFN-α against HCV, although the RNA level of HCV was unaffected by either the overexpression or absence of PCBP2 in R1b cells. RIP-qRT-PCR and RNA half-life further revealed that PCBP2 stabilizes the mRNA of STAT1 and STAT2 through binding the 3′Untranslated Region (UTR) of these two molecules, which are pivotal for the IFN-α anti-HCV effect. RNA pull-down assay confirmed that there were binding sites located in the C-rich tracts in the 3′UTR of their mRNAs. Stabilization of mRNA by PCBP2 leads to the increased protein expression of STAT1 and STAT2 and a consistent increase of phosphorylated STAT1 and STAT2. These effects, in turn, enhance the antiviral effect of IFN-α. These findings indicate that PCBP2 may play an important role in the IFN-α response against HCV and may benefit the HCV clinical therapy.

## Introduction

Hepatitis C virus (HCV) infection is a major world health problem with a worldwide carrier rate estimated at over 180 million people [1]. Persistently infected patients have a high risk of contracting liver diseases. HCV infection leads to chronic hepatitis in up to 60–80% of infected adults and then progresses to liver cirrhosis and hepatocellular carcinoma [2]. Interferon-α (IFN-α) has been shown to have beneficial effects in patients with chronic hepatitis C. IFN-α, especially pegylated IFN-α therapy, leads to a rapid decline in HCV RNA levels in serum, and long-term responses to therapy are marked by the sustained reduction of HCV RNA in the serum and liver, leading to resolution of chronic Infection [3], [4].

IFN-α possesses potent antiviral activity but does not act directly on the virus or replication complex. Rather, it acts by inducing IFN-stimulated genes (ISGs), that establish a non-virus-specific antiviral state within the cell [5]. In brief, circulating IFN-α binds to IFN cell surface receptor subunits. The binding leads to the activation of the receptor associated Janus-activated kinase 1 (Jak1) and tyrosine kinase 2 (Tyk2) [6], [7]. The activated kinases phosphorylate the signal transducer and activator of transcription proteins 1 and 2 (STAT1 and STAT2). The activated STAT1/2 complex combines with IFN-regulatory factor 9 (IRF-9) to form a complex that binds to the IFN-stimulated response elements on cellular DNA, which leads to the expression of the multiple ISGs [8]. Although IFN-α has been widely used to treat infected patients, relapses are frequent even with the combination of ribavirin, and more than half of those undergoing therapy remain fail to clear the virus [9]. The factors responsible for the persistence of HCV and the mechanisms by which HCV eludes the active immune response have yet to be identified.

A number of the cellular proteins have been reported to participate in the life cycle of HCV, such as cyclophilins [10], [11], which are essential for HCV replication and processing kinetics at the NS5A-B site. As HCV is a positive-stranded RNA virus, cellular RNA binding proteins (RBPs) are of particular importance in its life cycle. RBPs have been shown to interact with the viral ciselements. These RNA binding proteins include PTB [12], [13], HuR [14] and La antigen [15], all of which have been shown to be involved in HCV IRES-mediated translation initiation or virus genome replication. Poly(C) binding protein 2 (PCBP2, hnRNP E2 or α-CP2) belongs to the hnRNP E family of RNA binding proteins that interact with a sequence-specific motif with single-stranded poly(C) tracts. In its cellular capacity, PCBP2 is involved in posttranscriptional controls [16], protein interactions [17] and decoy of microRNA [18]. Apart from its roles in maintaining mRNA stability and regulating translation [19], PCBP2 can also participate in the replication or translation of many RNA viruses, including poliovirus [20], [21], coxsackievirus [22], and rhinovirus [23], which are positive sense RNA viruses structurally similar to HCV, but which belong to the family *Picornaviridae*. Investigations have demonstrated that PCBP2 can bind to the 5′-UTR [24] and 3′-UTR [25] regions of the HCV genome. These results suggest a relationship between PCBP2 and HCV. However, conflicting data exist as to how PCBP2 influences the HCV life cycle. Some researchers have reported that PCBP2 is induced in Japanese Fulminating Hepatitis-1 (JFH-1) infected cells and required for JFH-1 replication [26]. Other studies report that PCBP2 and PTB can rescue the inhibition of HCV IRES-mediated translation *in vitro* [27], while other findings indicate that PCBP2 has no direct effect on HCV replication or IRES-mediated translation [24], [25], [28].

In this study, we used the classical HCV replicon cell model and discovered significant down-regulation of PCBP2 in an HCV-subgenome replicon containing Huh7.5.1 cells (R1b) compared to naïve Huh7.5.1 cells. Although we experimentally validated that PCBP2 had no direct effect on HCV as reported before [24], [25], we also observed that PCBP2 facilitates the antiviral activity of IFN-α against HCV. Further investigations indicate that PCBP2 stabilizes the mRNA of STAT1 and STAT2 through binding the C-rich tracts in the 3′UTR of the two pivotal IFN-α signaling pathway molecule. These findings suggest that PCBP2 plays an important role in the cellular anti-HCV immune response. Further knowledge of PCBP2 may benefit clinical HCV therapy.

## Results

### HCV non-structural proteins 4B and 5A inhibit the expression of PCBP2 in R1b cells harboring the HCV subgenome replicon

**Figure 1:**
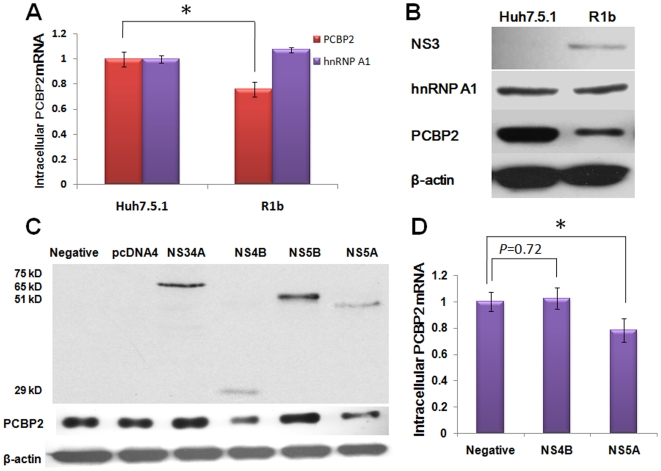
HCV non-structural proteins 4B and 5A mediate the down-regulation of PCBP2 in R1b cells with HCV subgenomic replicon. (A) qRT-PCR detection of PCBP2 mRNA level in Huh7.5.1 cells and R1b cells. The fold-relative enrichment was quantified with normalization to the GAPDH level. The mRNA level of PCBP2 in Huh7.5.1 replicon cells is significantly lower compared with Huh7.5.1 cells (*P*<0.05). (B) Western blotting indicates the down-regulation of PCBP2 protein level in R1b cells. (C) HCV non-structural protein NS4B and NS5A inhibit the expression of PCBP2 in Huh7.5.1 cells. NS4B and NS5A lead to the down-regulated expression of PCBP2 while other NS proteins demonstrate no effect. (D) qRT-PCR detection of PCBP2 mRNA level in Huh7.5.1 cells expressed HCV NS4B or NS5A proteins. NS4B demonstrate no effect on PCBP2 RNA level (*P* = 0.72), while NS5A can inhibit approximately 22% of PCBP2 RNA level. Each bar represents the average of triplicate data points with the standard deviation represented as the *error bar*. *P<0.05 versus negative control.

Sustaining viral replication with an HCV replicon in hepatoma cells requires many host factors. Several RBPs are believed to be essential in the replication and translation of HCV. We screened the RNA expression level of RBPs by qRT-PCR. Most RBPs were up-regulated or invariable (data not shown), while the mRNA level PCBP2 presented an approximately 25% decrease in R1b cells compared with Huh7.5.1 cells (Fig. 1A). Western blotting verified that PCBP2 protein levels were also significantly down-regulated in R1b cells (Fig. 1B). mRNA and protein levels of another RBP, hnRNP A1, were not significantly different between the two cells. As the main differences between the R1b cells and Huh7.5.1cells were the HCV subgenome replicon and sequentially expressed non-structural (NS) proteins, we speculated that HCV NS proteins contributed to the decrease of PCBP2 in R1b cells. To verify the observation,we constructed eukaryotic expression vectors for each HCV NS protein. NS3 was fused with NS4A because NS4A is a functional co-factor of NS3 and forms a noncovalent complex with NS3 [29]. The PCBP2 protein changes were detected by Western blotting after transfecting the recombinant plasmids into Huh7.5.1 cells (Fig. 1C). The PCBP2 protein level in cells with ectopic expression of NS4B and NS5A decreased compared with the pcDNA4 negative control, whereas no changes were observed in the NS34A- and NS5B-expressing cells and negative control compared with the untransfected cells. These results indicate that NS4B and NS5A may contribute to the inhibition of PCBP2 in R1b cells.

We further measured the PCBP2 mRNA level in cells with ectopic expression of NS4B and NS5A by qRT-PCR. NS4B demonstrated no effect on PCBP2 RNA level, but NS5A could lower PCBP2 RNA level (Fig. 1D) by approximately 22%. These results suggest that NS4B may post-transcriptionally regulate PCBP2 while NS5A may function a transcriptional regulation.

### IFN-α restores the expression level of PCBP2 in R1b cells after inhibiting the expression of the HCV replicon

**Figure 2:**
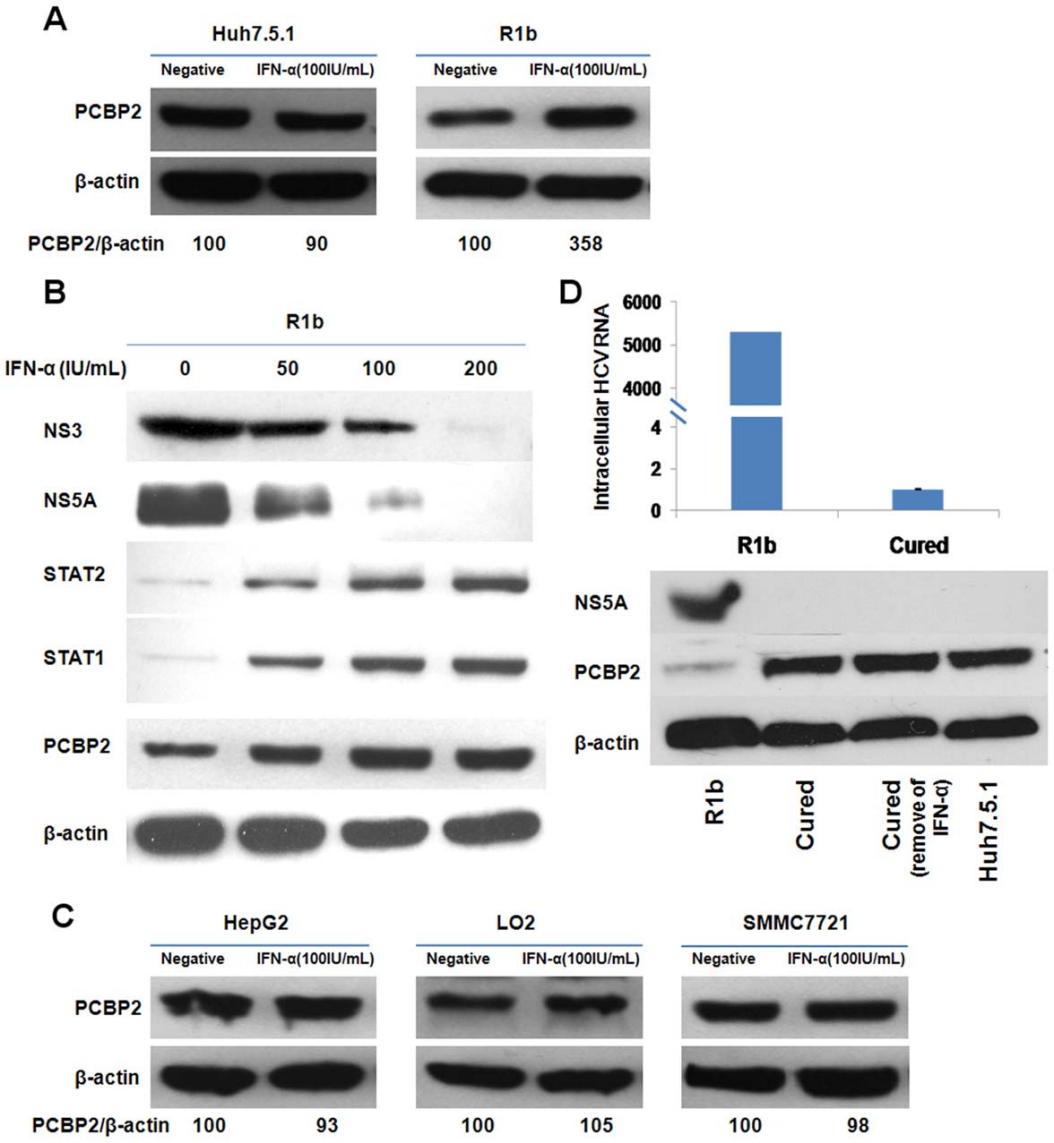
IFN-α restores the expression level of PCBP2 in R1b cells after inhibiting the expression of HCV replicon. (A) Western blotting analysis indicated prominent up-regulation of PCBP2 in R1b cells after treated with 100 IU/mL IFN-α, while no significant differences were noted in Huh7.5.1 cells. (B) The restoration of the expression of PCBP2 is IFN-α dosage dependent and due to inhibition of HCV NS proteins. IFN-α doses of 50, 100 and 200 IU/mL were used to treat R1b cells for 6 hours. Western blotting was performed to detect the expression of proteins. (C) PCBP2 protein level in other hepatocyte-derived cells after treatment of 100 IU/mL IFN-α. The PCBP2 level was unchanged, similar to that observed in Huh7.5.1 cells. (D) The protein level of PCBP2 was restored in cured R1b cells. The PCBP2 level did not return to a low level when IFN-α was removed from the media for a week.

HCV replicons are sensitive to the antiviral program induced by IFN-α. HCV protein and replicon RNA levels are reduced after treatment with IFN-α in a dose-dependent manner [30]. To verify if the inhibition of HCV protein can restore the expression of PCBP2 in R1b cells, 100 IU/mL IFN-α was used to treat R1b cells for 6 hours. After treatment with IFN-α, the PCBP2 level in R1b cells increased to over 3.5-fold compared to untreated cells (Fig. 2A) and the increase showed an IFN-α dose-dependent manner (Fig. 2B). A slight decrease was observed in Huh7.5.1 cells (Fig. 2A). Western blotting indicated that NS3 and NS5A expression was dramatically reduced in cells treated with IFN-α (concentration from 50 IU/mL to 200 IU/mL), whereas untreated control cells produced large amounts of HCV proteins (Fig. 2B). STAT1 and STAT2 were used as markers to assess the biological activity of the IFN-α preparation, and their expression were correspondent with IFN-α dosage (Fig. 2B). To exclude the possibility the lack of significant difference in PCBP2 was a peculiarity of Huh7.5.1 cell, three additional hepatocyte-derived cell lines (HepG2, SMMC7721 and LO2) were treated with IFN-α. The PCBP2 protein levels were unchanged in those cells as observed with the Huh7.5.1 cells (Fig. 2C).

When the R1b cells were treated with 1000 IU/mL IFN-α for two weeks to obtain cured cells, complete clearance of HCV RNA and proteins had occurred in the cells, and the PCBP2 levels were restored to the same level as observed in non-infected Huh7.5.1 cells and did not recede to the lower levels, even if IFN-α was removed from the media (Fig. 2D). These results demonstrate that IFN-α can restore the normal PCBP2 level in R1b cells by relieving the inhibition of the HCV NS proteins through clearance of HCV from the cells.

### PCBP2 facilitates the antiviral activity of IFN-α against HCV *in vivo*

**Figure 3:**
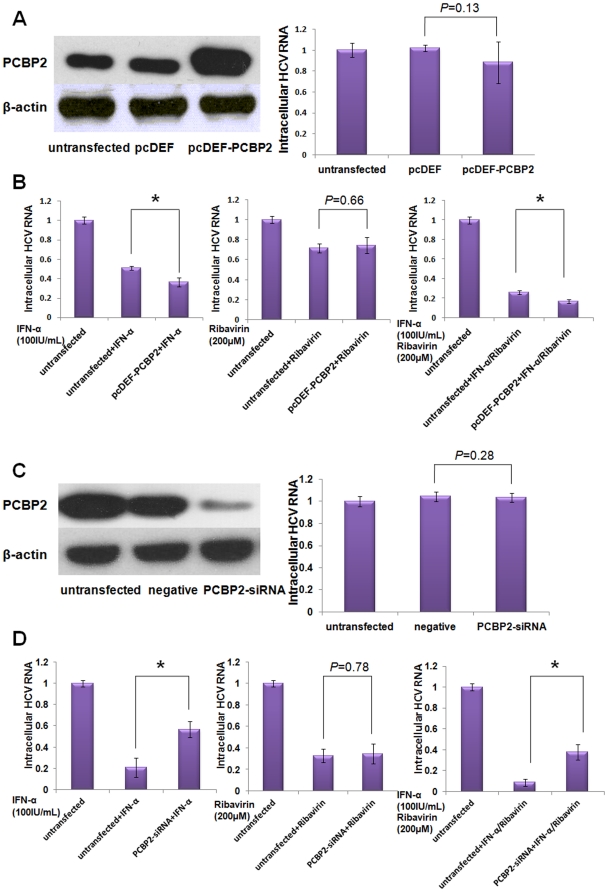
PCBP2 enhances the antiviral activity of IFN-α against HCV. R1b cells were transfected with pcDEF-PCBP2 or PCBP2 siRNA. 48 hours after transfection, the cells were treated with 100 IU/mL IFN-α, 200 µM ribavirin or a combination of the two, for 12 hours. Western blotting was used to check the overexpression or knockdown of PCBP2. qRT-PCR was performed to measure the HCV RNA level in the treated cells. (A) Overexpression of PCBP2 alone did not have an impact on the HCV RNA level (*P* = 0.13). (B) Overexpression of PCBP2 enhanced the inhibitory effects of IFN-α against HCV but did not influence the antiviral effect of ribavirin. (C) Knockdown of PCBP2 alone did not have an impact on HCV RNA level (*P* = 0.28). (D) Knockdown of PCBP2 impaired the inhibition effect of IFN-α against HCV but did not influence the antiviral effect of ribavirin. Each bar represents the average of triplicate data points with the standard deviation represented as the *error bar*. *P<0.05 versus negative control.

We have found that PCBP2 expression changes in R1b cells, but what is the relationship between PCBP2 and HCV? To illuminate that relationship, PCBP2 was overexpressed in R1b cells (Fig. 3A, left panel). qRT-PCR was performed to measure the HCV RNA level in cells. The HCV RNA level was unaffected by PCBP2 overexpression compared with the negative control and untransfected cells (Fig. 3A, right panel). However, when the PCBP2-overexpressed R1b cells were treated with IFN-α for 12 hours, HCV RNA level decreased over 15% compared to untransfected cells (Fig. 3B, left panel). Cells treated with IFN-α combined with ribavirin presented an accordant tendency similar to treatment with IFN-α alone (Fig. 3B, right panel). Ribavirin monotherapy did not demonstrate any significantly different influence in the PCBP2-overexpression cells compared with the untransfected cells (Fig. 3B, middle panel).

To confirm the role of PCBP2 on the antiviral activity of IFN-α against HCV, we performed PCBP2 knockdown. PCBP2 siRNA was transfected into the R1b cells. An unrelated siRNA acted as a negative control. Western blotting analysis revealed that the siRNA depleted PCBP2 proteins over 90% compared with the negative control and untransfected cells (Fig. 3C, left panel). As expected, HCV RNA levels were stable in PCPB2-depleted cells (Fig. 3C, right panel). However, depletion of PCBP2 also apparently hampered the activity of IFN-α against HCV. HCV RNA reduction in PCBP2-depleted cells decreased approximately 35% compared with untransfected cells (Fig. 3D, left panel). The IFN-α and ribavirin combination results and ribavirin monotherapy results further confirmed that PCBP2 affects IFN-α but has no effect on ribavirin (Fig. 3D, middle and right panel). Taken together, these results indicate that PCBP2 plays an important role in the anti-HCV effects.

### PCBP2 binds STAT1 and STAT2 mRNA and up-regulates the expression of the two signal molecules

**Figure 4:**
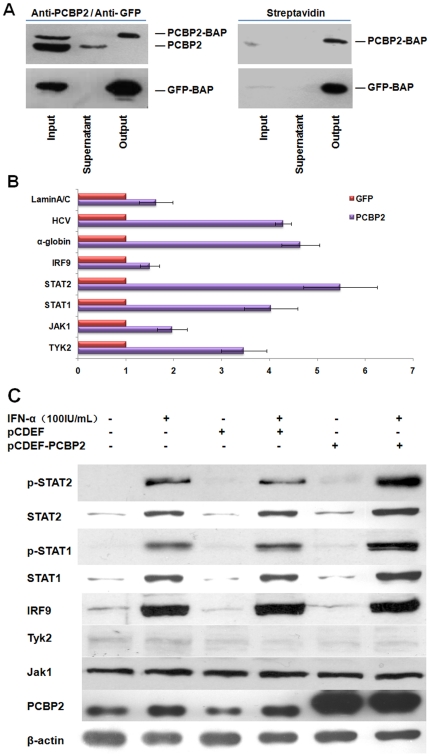
PCBP2 enhances the effect of IFN-α through binding STAT1 and STAT2 mRNA and up-regulates the expression of the two signal molecules in IFN-α pathway. RIP assay and targeted qRT-PCR confirmed the enrichment of different IFN-α signal pathway factor mRNAs in the PCBP2-RNA complex. (A) Detection of the PCBP2-RNA complex precipitated with streptavidin beads using anti-PCBP2 or anti-GFP antibody and streptavidin by Western blotting. (B) Targeted qRT-PCR was carried out on RNA isolated from RNP complexes precipitated in RIP assay. Analysis of mRNAs isolated from a GFP RIP reaction was carried out in parallel as nonspecific binding. α-globin was used as positive control. The enrichment of each mRNA was compared to the GFP control. Each bar represents the average of triplicate data points with standard deviation represented as the *error bar*. (C) PCBP2 up-regulated the expression of STAT1 and STAT2 after the treatment of IFN-α while other factors remained intact. The R1b cells were transfected with pcDEF vector or pcDEF-PCBP2. Forty-eight hours after transfection, the cells were treated with 100 IU/mL IFN-α and paired with untrasfected cells. Next, 6 hours later, the cell lysates were assessed by Western blotting.

To elucidate how PCBP2 facilitates the biological activity of IFN-α, we performed RNA Immunoprecipitation (RIP) to check if any IFN-α signaling pathway factors are involved in the substrates of PCBP2. Using streptavidin-sepharose beads, a complex of biotinylated proteins with binding RNA could be isolated from total cell lysate. Western blotting revealed that the RNP complex containing biotinylated PCBP2 and GFP could bind to the beads and be enriched (Fig. 4A). qRT-PCR was performed to check the enrichment of binding RNA. GFP presents nonspecific binding of RNA. α-globin and γ-globin served as positive and negative controls, respectively [31]. Lamin A/C represented an unrelated mRNA [32]. The relative mRNAs levels were compared with the RNA isolated from the PCBP2 RIP and GFP RIP by targeted qRT-PCR and normalized to the γ-globin mRNA levels. α-Globin mRNA was enriched by nearly 5-fold in the PCBP2-RIP compared to the GFP control. The enrichment of STAT1 and STAT2 mRNAs corresponded to that of α-globin. However, IRF9, JAK1 and TYK2 levels were significantly lower than that of α-globin (Fig. 4B). Notably, we detected that HCV RNA was enriched in the PCBP2 RNP complex. The results indicate that the STAT1 and STAT2 mRNAs may be the substrates of PCBP2 and the effect on IFN-α may relate to the influence on STAT1 and STAT2.

Whether the binding of mRNA to PCBP2 will lead to the expression of proteins remain to be verified. PCBP2 was overexpressed in R1b cells subsequently with treatment of IFN-α. The expression of IFN-α pathway factors were detected by Western blotting. IFN-α induced the expression of IRF9, STAT1 and STAT2, but exerted no influence on JAK1 and TYK2. Overexpression of PCBP2 distinctly increased the STAT1 and STAT2 protein levels in comparison to a negative control, while no significant differences were observed with respect to IRF9, JAK1 and TYK2 (Fig. 4C). It should be noted that there was also a consistent increase in phosphorylated STAT1 and STAT2, which may due to the increase of total proteins. These data indicate that PCBP2 binds the STAT1 and STAT2 mRNAs and up-regulates their protein levels.

### The C-rich tracts in the 3′UTR of mRNA of STAT1 and STAT2 are PCBP2 binding sites

**Figure 5:**
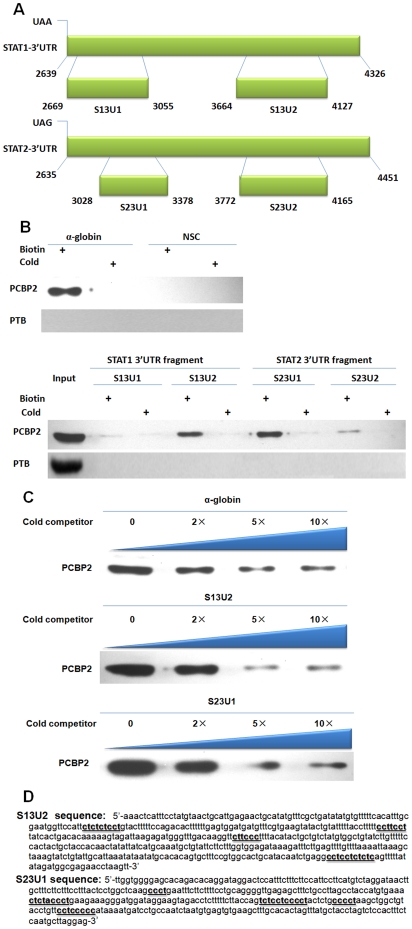
The C-rich tracts in the 3′UTR of mRNA of STAT1 and STAT2 are PCBP2 binding sites. (A) Fragments of the STAT1 and STAT2 3′-UTR used in synthesis of sense RNA probe *in vitro* and sequences position in the 3′-UTR. (B) RNA pull-down assay using the four probes of STAT1 and STAT2. Cytoplasmic extracts from 100 IU/mL IFN-α treated R1b cells were incubated with biotin-labeled RNA probes. The mixture was pulled down by streptavidin beads. The resulting complexes were separated by SDS-PAGE and detected by Western blotting. A pGEM-3zf vector sequence was utilized as a nonspecific control (NSC), and the α-globin 3′-UTR was utilized as a positive control. The bottom panel depicts the assay results with the RNAs set of fragments shown in *A*. (C) 2-fold, 5-fold and 10-fold of excess unlabeled cold probes were added to compete with the labeled probes for competition analysis. The results indicate that 5-fold cold competitors of S13U2 and S23U1 probe exhibited strong competition. (D) The nucleotide sequences of S13U2 and S23U1. The CU-rich patches are underlined.

In many cases, PCBP2 functions through binding the 3′UTR of target mRNA. As we found the STAT1 and STAT2 mRNA interacted with PCBP2, we decided to test which regions of STAT1 and STAT2 3′-UTR interacted with PCBP2 by RNA pull-down assay. Four fragments located in the 3′UTR of STAT1 and STAT2 were inserted into a pGEM-3zf vector containing a T7 promoter. These fragments were predicted as potential binding sites by sequence analysis as they contained C-rich tracts harboring the core binding sequence of PCBP2 [33]. The maps of these regions are shown in Fig. 5A. The biotin-labeled RNA probes corresponding to each fragment were synthesized *in vitro* with T7 RNA polymerase. The pGEM-3zf vector sequence and the α-globin 3′-UTR were used as a non-specific control (NSC) and a positive control, respectively. The proteins pulled down by the probes were analyzed by Western blotting using an anti-PCBP2 antibody. PCBP2 was not detected in the NSC pull-down complex. In contrast, the α-globin mRNA showed a strong binding of PCBP2 (Fig. 5B, upper panel). As for the four probes, the S13U2 and S23U1 probes presented significant linking products accordant with α-globin (Fig. 5B, lower panel). S13U1 did not demonstrate binding of PCBP2, and S23U2 only had very faint non-reproducible linking products. PTB is an important HCV-related RBP that does not contain consensus PCBP2 binding sequences in its mRNA. We assayed PTB with the RNA pull-down complex, and the results indicated that PTB did not demonstrate any binding of the 3′UTR of STAT1 and STAT2. These data demonstrate that PCBP2 binding to the 3′UTR of STAT1 and STAT2 is specific.

To further confirm whether the binding of PCBP2 to S13U2 and S23U1 is a specific result, a competition assay was performed. Non-biotin labeled homologous probes (cold competitors) were added to compete with biotin-labeled probes in PCBP2 interaction. The cold competitors exhibited strong competition only at 5-fold molar excess (Fig. 5C). Therefore, we conclude that the major binding determinant of PCBP2 resides in the S13U2 and S23U1 fragments. The sequence of the two fragments is indicated in Fig. 5D. The C-rich regions (underlined) bear a striking resemblance to the C-rich motifs previously identified as the PCBP2 binding site in human α-globin mRNA [31].

### PCBP2 enhances the expression of STAT1 and STAT2 through stabilizing their mRNA

**Figure 6:**
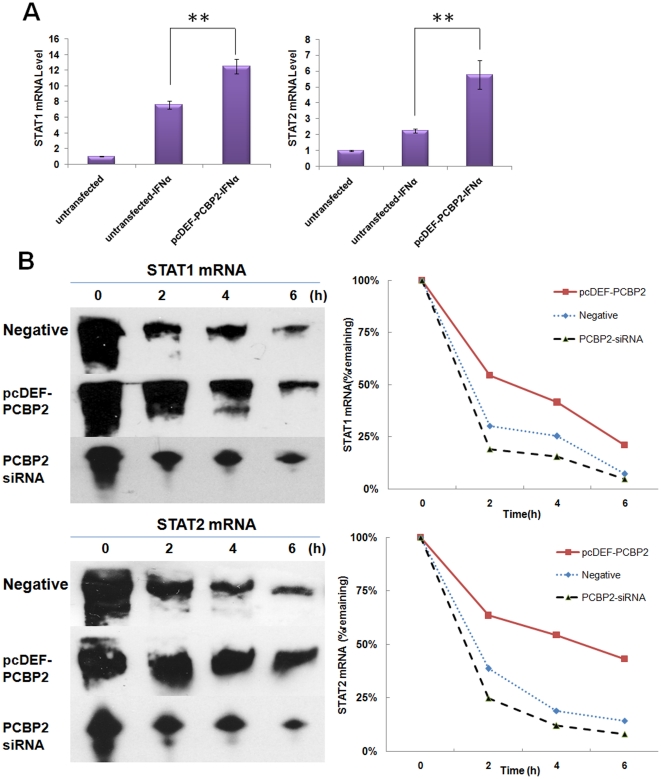
The STAT1 and STAT2 mRNA is stabilized in cells overexpressing PCBP2. (A) qRT-PCR was carried out to measure mRNA level of STAT1 and STAT2 after 6 hours of IFN-α treatment. R1b cells were untransfected or transfected with pcDEF-PCBP2. Each bar represents the average of triplicate data points with the standard deviation represented as the *error bar*. **P<0.001 versus untransfection. (B) RPA and RNA half-life analysis was performed to monitor the STAT1 and STAT2 mRNA decay course. Two days after transfection, IFN-α was added and 6 hours later actinomycin D was added to inhibit transcription. Representative blots are shown in the left panel and the mRNA levels were quantified through densitometric scanning. For each set of RPAs, the band intensity at the 0 h time point was set to 100%, and the percentage of mRNA of remaining at the 2, 4 and 6 hour time point was plotted shown in the right panel.

PCBP2 enhances the protein level of STAT1 and STAT2, and it has been demonstrated that C-rich tracts in their 3′UTR are the binding sites of PCBP2. However, how PCBP2 affects mRNA remains to be elucidated. The interaction of PCBP2 proteins with the STAT1 and STAT2 mRNA suggests that control over their mRNA levels might be mediated by an effect on mRNA stability. qRT-PCR revealed an over 80% increase of STAT1 mRNA and a 150% increase of STAT2 mRNA in PCBP2-overexpressed cells compared with negative controls after treatment with IFN-α (Fig. 6A). A RPA was carried out to verify the mRNA decay course. RNA harvested at subsequent time points was quantified for STAT1 and STAT2 mRNA levels by RPA analysis. PCBP2 overexpression and knockdown were performed in R1b cells. A pcDEF vector and unrelated siRNA acted as negative controls, respectively. This analysis revealed that the rates- of STAT1 and STAT2 mRNA decay in negative control cells were similar, with an average half-life of approximately 1.4 hours and approximately 1.7 hours respectively. In contrast, PCBP2 overexpression resulted in prolongation of half-life to approximately 2.8 hours and approximately 4.9 hours, while knockdown of PCBP2 shortened their half-lives to approximately 1.1 hours and approximately 1.3 hours, respectively (Fig. 6B). This alteration in STAT1 and STAT2 mRNA stability in the PCBP2 -overexpression cells was consistent with the observed increase in their mRNA levels and protein. The mRNA stabilization encouraged by PCBP2 supports the up-regulation of STAT1and STAT2 proteins and accordingly the effect on IFN-α.

## Discussion

**Figure 7:**
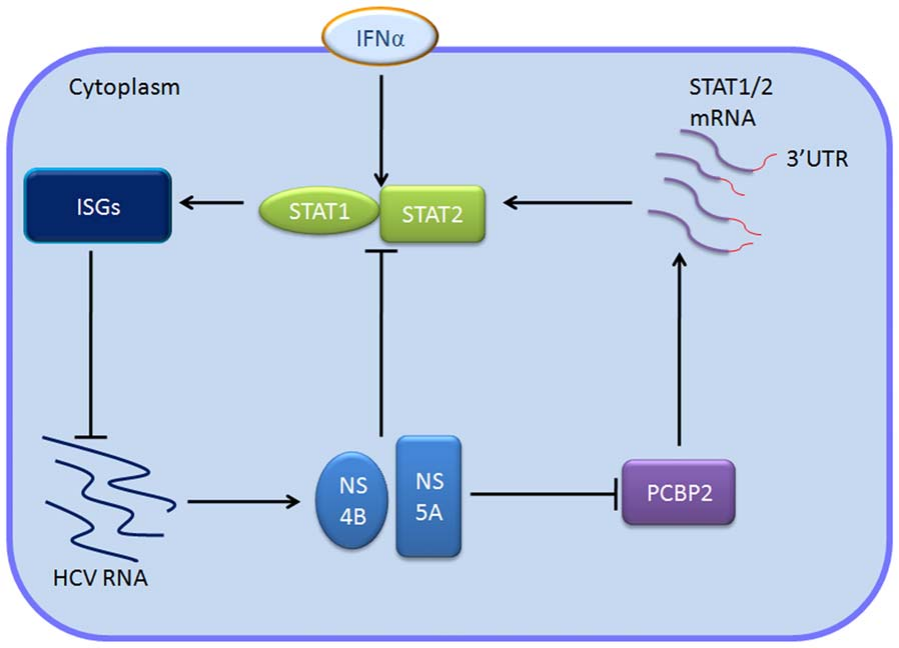
Model of PCBP2-modulating effect of IFN-α against HCV. PCBP2 enhances the antiviral activity of IFN-α through stabilizing the mRNA of STAT1 and STAT2. This accordingly, increases the protein level of the two molecules. The protein level increase also enhances the effect of IFN-α against HCV.

Cells harboring HCV replicons provide the basis for a long-sought cellular system that allows detailed molecular studies of HCV and the development of antiviral drugs [34]. In our work we utilized the classical HCV replicon cellular system to examine the molecular mechanism by which PCBP2 and IFN-α inhibit HCV. The major novel findings in this work include the followings: PCBP2 facilitates the antiviral activity of IFN-α against HCV, STAT1 and STAT2 mRNA serve as binding substrates of PCBP2 and are stabilized by PCBP2. Here, we propose a model where PCBP2 modulates the effects of IFN-α against HCV (Fig. 7). In R1b cells, the HCV subgenomic replicon abundantly expresses viral NS proteins. These NS proteins impair cellular molecules as an attempt to resist host immune responses [35]. The NS-mediated inhibition of PCBP2, as discovered in our work and verified by ectopic expression of NS proteins in naïve Huh7.5.1 cells (Fig. 1), further supports the idea of NS proteins serving as anti-host immunity proteins Investigations have supported the point that viral NS proteins can degrade STAT1 [36] and STAT2 [37], or suppress STAT1 phosphorylation [38], [39], which may explain the extremely low expression levels of STAT1 and STAT2 in R1b cells (Fig. 2B). When exogenous IFN-α is added to treated R1b cells, STAT1 and STAT2 are induced and promote downstream ISGs expression that, in turn, inhibits HCV expression. This relieves the inhibition of PCBP2 in R1b cells (Fig. 2A). PCBP2 can facilitate the antiviral activity of IFN-α against HCV (Fig. 3), which explains the inhibition of PCBP2 exerted by HCV NS proteins. RIP-qRT-PCR demonstrates the mRNA of STAT1 and STAT2 are enriched in the PCBP2 RNP complex, which implies that they are binding substrates of PCBP2 (Fig. 4B). Further studies confirm the poly-C tracts in the 3′UTR of STAT1 and STAT2 mRNAs are a PCBP2 binding site (Fig. 5). The stability of the STAT1 and STAT2 mRNAs is increased as evidenced by elongation of mRNA half-life (Fig. 6B) and leads to the up-regulated protein level of the two signaling molecules (Fig. 4C). As STAT1 and STAT2 play an indispensable role in antiviral immunity against HCV expression, PCBP2 accordingly facilitates the effects of IFN-α against HCV. In summary, these factors interact with each other in HCV replicon cells and form a loop pathway (Fig. 7).

PCBP2 has been demonstrated to influence the life cycles of positive-stranded RNA viruses [21], [22], but no direct effects has been revealed in terms of an interaction with HCV. The binding of PCBP2 to the HCV genome was verified in our studies, but the mechanism and biological function of underlining the PCBP2-HCV interaction remain to be explored. As many RBPs are employed by HCV to promote translation or replication generally through direct binding with HCV 5′- or 3′-UTR regions, the PCBP2 facilitation of the effect of IFN-α against HCV illuminates new RBPs characteristics that mediate the life processes of HCV.

Although ectopic expression of HCV NS proteins demonstrates inhibition of PCBP2 in R1b cells, the regulation of PCBP2 is still not clear. No evidence exists demonstrating how HCV NS proteins can degrade PCBP2 mRNA or exert translation repression. A BCR/ABL-MAPK^ERK1/2^-PCBP2 pathway was established in myeloid chronic myelogenous leukemia blast crisis (CML-BC) progenitors [40]. In the cells, PCBP2 expression was induced by BCR/ABL in a dose- and kinase-dependent manner through constitutive activation of MAPK^ERK1/2^. It is speculated that HCV may impair this pathway and consequently inhibit the expression of PCBP2. However, whether the pathway plays a role in hepatoma cells harboring HCV replicon remains to be examined.

The two most intensively studied posttranscriptional controls mediated by PCBP2 are mRNA stabilization and translational modulation. The initial mRNA identified as a PCBP2 stabilization target was human α-globin mRNA. The 3′-UTR of this mRNA led to the identification of the corresponding RNP complex [31] that forms at this site. In our experiments, α-globin was utilized as a positive control and demonstrated a robust interaction with PCBP2. STAT1 and STAT2 mRNA were verified to be involved in PCBP2 containing complexes with long half-lives. These findings lead to a model in which 3′-UTR–PCBP2 complexes can serve as general determinants of mRNA stability. Previous studies have revealed that additional mRNAs, such as those encoding tyrosine hydroxylase [41] and lipoxygenase have closely related [42], if not identical, PCBP containing 3′-UTR complexes. It is also known that PCBP2 mRNA itself is represented in PCBP2-RNP complexes, suggesting autoregulatory control of PCBP2 expression [31]. However, as the corresponding mechanisms involved in the proposed autoregulation of PCBP2 expression are still to be elucidated, additional experiments are required to prove that this mechanism is used by HCV to reduce PCBP2 expression.

Although IFN-α was applied as a treatment of HCV as a crucial mediator of the innate antiviral immune response for decades, treatment with IFN-α alone has achieved only modest success, and half of the infected individuals with chronic disease do not achieve sustained clearance of HCV [5]. Abnormalities in the downstream actions of IFN-α activity mediated by HCV have been indicated in several studies. These findings provide a biological basis for correlations between the HCV NS proteins and the lack of response to IFN-α therapy [35], [36], [38], [39]. However, the cellular factors participating in IFN-α antiviral response are still unclear. Identification of the host factors that can predict sustained virological response (SVR) will undoubtedly improve the treatment of hepatitis C. We have found that PCBP2 plays an important role in influencing anti-HCV IFN-α. High levels of PCBP2 result in a more powerful effect of IFN-α in HCV replicon cells, while the absence of PCBP2 impairs that effect. If the PCBP2 level corresponds to the virological response of IFN-α in HCV clinical therapy, it may be a prospective marker for clinical diagnosis for using IFN-α.

We have demonstrated that STAT1 and STAT2 were involved in the action of PCBP2 on IFN-α. The mRNA of some ISGs, such as IRF9, JAK1 and TYK2, were also assayed in the RNP complex obtained in RIP assay. Despite their mRNA being enriched in the PCBP2 complex compared to the GFP complex, their protein levels were not affected by PCBP2. The binding of their mRNA to PCBP2 may represent nonspecific binding. Other important ISGs, for example PRKR, Mx1, OAS2 and ADAR1, are important for the antiviral activity of IFN-α [43]. Whether PCBP2 could affect their mRNA and, correspondingly the antiviral activity of IFN-α is of interest and remains to be investigated.

In conclusion, our work reveals a new mechanism elucidating how RNA-binding proteins interact with HCV, and more importantly, these findings suggest the inclusion of clinically relevant PCBP2 in the HCV therapy.

## Materials and Methods

### Cell culture and cell lines

Huh7.5.1 human hepatoma cells, kindly provided by Francis Chisari (Scripps Research Institute, La Jolla), were maintained in complete DMEM supplemented with 2 mM L-glutamine, 100 U/ml of penicillin, 100 mg/ml of streptomycin and 10% FBS. HepG2 (ATCC), SMMC7721 [44] and LO2 [45] were maintained in MEM and RPMI1640 medium with the same supplements described above, respectively. R1b cells were HCV 1b subgenome replicon harboring Huh7.5.1 cells developed in our laboratory as described previously [34] and maintained with an additional 500 µg/mL G418 supplement. To prepare cured cells [46], the R1b cells were treated with 1000 IU/ml of IFN-α in the absence of G418. The treatment was continued for 2 weeks with the addition of IFN-α at 3-day intervals. qRT-PCR confirmed the clearance of HCV RNA in these cured cells.

### Ectopic expression of HCV NS proteins

HCV NS3-, NS4A-, NS4B-, NS5A- and NS5B-encoding fragments were amplified from a plasmid containing the HCV genome and inserted into a pcDNA4-myc vector (Invitrogen), respectively. The plasmids were transfected into Huh7.5.1 cells using Lipofectamine 2000 (Invitrogen) according to the manufacturer's instructions. The protein lysates were analyzed by Western blotting using anti-myc tag antibody to detect the expression of NS proteins.

### IFN-α treatment of cells with overexpression or knockdown of PCBP2

R1b cells were transfected with either siRNA targeting PCPB2 [47] or a pcDEF-PCBP2 plasmid using Lipofectamine 2000. Non-related siRNA and pcDEF-myc vector were used as negative controls. The siRNA sequences designed to silence PCBP2 gene expression and a non-related control were 5′-GGCCTATACCATTCAAGGA-3′ and 5′–ACGUGACACGUUCGGAGA ATT–3′, respectively. Twenty-four hours after transfection, the cells were treated with 100 IU/mL IFN-α or 200 µM Ribavirin and were harvested 6 hours later. The cells were lysed in cell lysis buffer for western blotting analysis to examine the expression levels of various proteins or in Trizol (Invitrogen) for qRT-PCR analysis to check the HCV RNA level.

### Western blotting

Western blotting was performed following a previously described method [32]. Total protein was extracted with lysis buffer (150 mM NaCl, 1% NP40 and 50 mM Tris–HCl, pH 8.0) supplemented with protease inhibitors (2 µg/ml leupeptin, 2 µg/ml pepstain, 2 µg/ml aprotinin and 2 µg/ml PMSF). The lysate was separated by 10% sodium dodecyl sulfate polyacrylamide gel electrophoresis, and the gel was transferred onto nitrocellulose membrane. Antibodies against corresponding proteins were used in proper dilution to detect the protein level. Specific primary antibodies were obtained from the following sources: The PCBP2 antibody obtained from Aviva. The STAT1, STAT2, IRF9, JAK1, TYK2 and hnRNP A1 antibodies were from Santa Cruz Biotechnology. The HCV NS3 and NS5A antibodies were from Virostat. phosphorylated-STAT1 and phosphorylated-STAT2 were obtained from Cell Signaling Technology. The PTB antiserum was developed in our laboratory.

### qRT-PCR

qRT-PCR was performed following a previously described method [32]. In brief, qRT-PCR analysis was performed in an ABI 7500 Sequence Detection System (Applied Biosystems) as follows: 1 minute at 95°C, 40 cycles of 10 seconds at 95°C and 34 seconds at 60°C. The primers are described in detail in Table S1. The fold-relative enrichment was quantified with normalization to the GAPDH level.

### RNA immunoprecipitation (RIP)

RIP was performed following a previously described method [48]. In brief, a tandem affinity purified tag, Biotin-acceptor-peptide (BAP), was fused to the 5′-terminal of the encoding sequences of PCBP2 and GFP, which were co-transfected with BirA (biotin ligase) into R1b cells using Lipofectamine 2000. Following that, 1 mM biotin and 100 IU/mL IFN-α were added in the culture medium immediately after transfection. Forty-eight hours later, the biotinylated-tagged proteins and RNA complex were isolated from cell lysate using high affinity streptavidin-sepharose beads (GE Healthcare). RNP was eluted from the beads and the RNA was purified from RNP using Trizol. cDNA was synthesized from the isolated RNA and then subjected to qRT-PCR for detection of RNA enrichment using the primer sets described in Table S1.

### *In vitro* transcription

The sense RNA probes S13U1, S13U2, S23U1, S23U2 and α-globin were synthesized by *in vitro* transcription using T7 RNA polymerase (Takara). A nonspecific probe was synthesized from the pGEM vector. To generate biotin-labeled RNA transcripts, 1 µg of each DNA template was added to *in vitro* transcription reactions containing biotin-UTP (Roche). Non-labeled competitor RNA probes were synthesized with unlabeled UTP. After purification and removal of unincorporated nucleotides [49], the RNA was resuspended in Buffer A (50 mM potassium acetate, 3 mM magnesium acetate, 2 mM dithiothreitol, 20 mM HEPES, pH 7.4). Anti-sense RNA probes were synthesized using SP6 RNA polymerase (Takara) following the same methods as used for the sense RNA probes.

### Biotin-RNA pull down

The biotin-labeled sense RNA probes were synthesized *in vitro* using T7 RNA polymerase. Cytoplasmic cell extracts were isolated from R1b cells treated with 100 IU/mL IFN-α. RNA affinity capture was subsequently carried out with streptavidin-sepharose beads as described previously [50]. The elutes were separated on 10% SDS-PAGE for PCBP2 detection by Western blotting. For competition analysis, 2-fold, 5-fold and 10-fold of excesses unlabeled cold competition RNAs were added to compete with the labeled RNAs.

### mRNA half-life and RNase protection assay (RPA)

RPA was performed as reported in detail previously [51] with some minor modifications. Briefly, biotin-labeled antisense probes were synthesized in vitro with SP6 RNA polymerase (Takara) from DNA templates constructed for an RNA pull-down assay. Next, 100 IU/mL IFN-α was added to the media at 2 days post-transfection and 4 hours later Actinomycin D (Sigma, 5 µg/ml) was added. The total RNA was collected at subsequent 0, 2, 4 and 6 hour time points. Ten micrograms of total RNA from each time point sample was cohybridized with the antisense probes at 45°C overnight. The excess probes and unhybridized RNA were eliminated by treatment with RNase A/T1 (Ambion). The precipitated protected RNA fragments were resolved on a 6% polyacrylamide gel containing 8 M urea and then electrophoretically transferred to a positively charged nylon membrane. The membrane was used directly to detect the protection fragments using a Chemiluminescent Nucleic Acid Detection Kit (Pierce) according to the manufacturer's instructions. Quantitation was performed via densitometric scanning with Quantity One software (BioRad).

### Statistical analysis

All the experiments were performed three times and the data are presented as the mean ±3 standard deviations (SD). The results were analyzed using a paired Student's *t* test to determine the significance of observed differences between the control and individual treatment values. *P*<0.05 was regarded as statistically significant.

## Supporting Information

Table S1 RIP-qRT-PCR Primers. The primer sets of mRNAs detected in qRT-PCR assay are listed in the table.
(DOC)
