# Supplementary material for: PCBP2 Enhances the Antiviral Activity of IFN-α against HCV by Stabilizing the mRNA of STAT1 and STAT2
Source: PLoS One. 2011 Oct 11;6(10):e25419. doi: 10.1371/journal.pone.0025419 (PMC3191149; doi:10.1371/journal.pone.0025419)
Supplement: Table S1 — RIP-qRT-PCR Primers. The primer sets of mRNAs detected in qRT-PCR assay are listed in the table. (DOC) [file pone.0025419.s001.doc]

**Table S1. RIP-qRT-PCR P**rimers

| **RNA** | **Primer Set (forward, reverse)** |
| --- | --- |
| HCV  TYK2  JAK1  STAT1  STAT2  IRF9  α-globin  γ-globin  Lamin A/C | 5’-TAGCGGCAAGAGCACTAAGG-3’, 5’-ACGGGTTCAGGACAAGCAC-3’  5’-CCTGCTGGAGCCATTTGTG-3’, 5’-ACTGTGAGGATCAGGCGGTAG-3’  5’-ACCAGGATGCGGATAAATAATG-3’, 5’-CAAAGTTTCCAAGGTAGCCAAG-3’  5’-GAACTGGTTCACTATAGTTGCGG-3’, 5’-TGTGATAGGGTCATGTTCGTAGG-3’  5’-ACACCCTCCCTGTGGTGATTA -3’, 5’-GTTCTGAAGGTTTGGGCTGAG-3’  5’-GAGCCACAGGAAGTTACAGACAC-3’, 5’-TGCTGCTCCCAATGTCTGAA-3’  5’-TCCCCACCACCAAGACCTAC-3’, 5’-CCTTAACCTGGGCAGAGCC-3’  5’-AGGAGGACAAGGCTACTATCACAA-3’, 5’-CCTCCAGCATCTTCCACATTC-3’  5’-AAGTCCAATGAGGACCAGTCC-3’, 5’-CGGTAAGTCAGCAAGGGATC-3’ |
